# Palonosetron/Methyllycaconitine Deactivate Hippocampal Microglia 1, Inflammasome Assembly and Pyroptosis to Enhance Cognition in a Novel Model of Neuroinflammation

**DOI:** 10.3390/molecules26165068

**Published:** 2021-08-21

**Authors:** Reem A. Mohamed, Dalaal M. Abdallah, Amany I. El-brairy, Kawkab A. Ahmed, Hanan S. El-Abhar

**Affiliations:** 1 Department of Pharmacology and Toxicology, Faculty of Pharmacy, October University for Modern Sciences and Arts, 26 July Mehwar Road Intersection with Wahat Road, 6th of October City, Giza 12451, Egypt; Ralia@msa.edu.eg (R.A.M.); aelbrairy@msa.eun.eg (A.I.E.-b.); 2 Department of Pharmacology and Toxicology, Faculty of Pharmacy, Cairo University, Κasr El-Aini Str., Cairo 11562, Egypt; Hanan.elabhar@pharma.cu.edu.eg; 3 Department of Pathology, Faculty of Veterinary Medicine, Cairo University, Giza 12211, Egypt; kawkababdelaziz@cu.edu.eg

**Keywords:** 5-HT3 receptor blocker, inflammasome, pyroptosis, caspase-1/IL-1β/IL-18, microglia, α7AChR

## Abstract

Since westernized diet-induced insulin resistance is a risk factor in Alzheimer’s disease (AD) development, and lipopolysaccharide (LPS) coexists with amyloid β (Aβ)1-42 in these patients, our AD novel model was developed to resemble sporadic AD by injecting LPS into high fat/fructose diet (HFFD)-fed rats. The neuroprotective potential of palonosetron and/or methyllycaconitine, 5-HT3 receptor and α7 nAChR blockers, respectively, was evaluated after 8 days of daily administration in HFFD/LPS rats. All regimens improved histopathological findings and enhanced spatial memory (Morris Water Maze); however, palonosetron alone or with methyllycaconitine promoted animal performance during novel object recognition tests. In the hippocampus, all regimens reduced the expression of glial fibrillary acidic protein and skewed microglia M1 to M2 phenotype, indicated by the decreased M1 markers and the enhanced M2 related parameters. Additionally, palonosetron and its combination regimen downregulated the expression of ASC/TMS1, as well as levels of inflammasome downstream molecules and abated cleaved caspase-1, interleukin (IL)-1β, IL-18 and caspase-11. Furthermore, ACh and 5-HT were augmented after being hampered by the insult. Our study speculates that blocking 5-HT3 receptor using palonosetron overrides methyllycaconitine to combat AD-induced neuroinflammation and inflammasome cascade, as well as to restore microglial function in a HFFD/LPS novel model for sporadic AD.

## 1. Introduction

Alzheimer’s disease (AD) is the most common cause of dementia among neurodegenerative disorders, with around 90% of cases having a sporadic non-familial type [1]. These patients usually present with more hippocampal volume loss, higher incidence of diabetes, obesity and circulatory disorders compared to the familial type [2]. Though the deposition of senile plaques of beta amyloid peptide (Aβ) and neurofibrillary tangles (NFTs) is inevitably linked to AD pathogenesis [3], these biomarkers are not the causative factors for the disease progression, but rather are downstream cues of unrelated triggers [4]. Lately, a possible role for the bacterial neurotoxin lipopolysaccharide (LPS), originating from the gastrointestinal flora or some dormant microbiomes, has been proposed in causing sporadic AD, since LPS was co-localized with beta amyloid peptide (Aβ) in brains of patients with sporadic AD. In experimental settings, LPS combined with focal cerebral ischemia or hypoxia produced amyloid-like plaques in rat cortexes [5]. 

LPS is known to trigger inflammation that partakes highly in the disease pathogenesis [6], a fact that supports the inflammatory neurodegenerative theory of non-familial AD [5]. In addition, both LPS and Aβ1-42 bind to the microglial Toll-like receptor (TLR)4 to activate the transcription factor nuclear factor-kappa B (NF-κB), which in turn reactivates the TLR4 to intensify the formation of Aβ1-42 and maintain microglia activation [5]. These interactions initiate a constellation of cellular alterations, including the activation of inflammasomes, which are cytosolic large multi-molecular neuroinflammatory signaling complexes that contribute to cognitive impairment [7]. This cascade can be elicited by pathogen- or danger- associated molecular patterns [8]. Notably, the NOD-like receptor family pyrin domain containing (NLRP)3 is the best described inflammasome that consists of the NLRP3 protein, the adapter protein apoptosis-associated speck-like protein (ASC)/target of methylation-induced silencing (TMS)1, and procaspase-1 [8]. Upon activation, procaspase-1 is cleaved to its active form caspase-1 that proteolytically activates interleukin (IL)-18 and IL-1β [8] that are mainly secreted by activated microglia [9].

Moreover, metabolic dysfunction has a documented impact on brain function causing dementia, including sporadic AD [10,11] and shares in cognitive impairment via several disturbed proceedings, such as the activated inflammasome [12], which participates in the altered cognition following consumption of high fat [13], and free fructose [14] diets. A high fat diet (HFD) administration also increases neuronal stress and causes insulin resistance (IR), which reduces glucose transportation into the brain, ultimately affecting the ability of the neurons to use glucose for energy [15]. Fructose administration, conversely, stimulates the synthesis of triglyceride to boost IR and promote gluconeogenesis [16] and accumulates in the brain to form advanced glycation end products, increases oxidative stress, and accelerates the progression of AD. Therefore, the combination of high fat and high fructose in the diet increases the risk factors for neurodegeneration [16]. Additionally, diabetes induced by a diet rich in saturated fat and/or fructose was reported to alter gut microbiota that may induce a state of endotoxemia via alteration of gut permeability [17,18] and even the blood–brain barrier (BBB) to impair cognition [19]. 

Undoubtedly, altered levels of neurotransmitters occur in AD, such as acetylcholine (ACh), but this is only the tip of the iceberg, since this widespread neuronal degeneration process affects many anatomical pathways, including the serotonergic system [20]. In a previous work [21], we have proven that tropisetron, a 5-hydroxytryptamin (5-HT)3 receptor blocker, elevates the hippocampal levels of 5-HT in experimental animals; besides this, 5-HT3 receptor blockers were previously recorded to possess neuroprotective and anti-inflammatory effects [22]. Apart from its 5-HT3 receptor blocker effect, tropisetron also exhibited a selective partial agonistic effect on α7 nicotinic acetylcholine receptors (α7nAChR) and attenuated Aβ-induced inflammatory and apoptotic responses in rats [23]. From the same class, palonosetron, the FDA-approved antiemetic, was reported to protect against surgical esophagitis [24] and to ameliorate preneoplastic colon damage through downregulating the expression of acetylcholinestrase to enhance synaptic ACh [25]. Moreover, the impact of maladaptive α7nAChR on cognitive disorders, such as AD, has long been reported [26,27]. Though the blockage of α7nAChR in normal rats by the selective blocker methyllycaconitine (MLA) has induced an in vivo model of cognitive impairment [28]; however, the interaction of increased fibrillary Aβ complexes with α7nAChR in AD rats contributed to synaptic dysfunction [26].

Due to the inadequacy of experimental models, a defective extrapolation between pre-clinical and clinical trials for the management of AD exists [4]. Hence, several trials have been carried out to develop animal models that relatively resemble the allied symptoms perhaps by using LPS alone or accompanied with ischemic or hypoxic injury to enhance its delivery to the brain [5,29] or by associating high fat feeding to rodents with transgenic models of AD [30,31]. Accordingly, our study has first verified a newly designed experimental model of neuroinflammation that resembles sporadic AD, using LPS in rats fed high fat/high fructose diet (HFFD); then, we have used this model to investigate the potential neuroprotective impact of the post-administration of palonosetron with or without methyllycaconitine (MLA), to assess the role of α7nAChR in the 5-HT3 blocker effect. Additionally, the possible involvement of the NLRP3 inflammasome cascade, microglia phenotypes, and the neurotransmitters ACh and 5-HT was also unveiled, as well as the effect of the chosen regimens on Aβ plaque formation, immune-reactivity of hypertrophied astrocyte, histopathological perturbations, and compromised cognitive function.

## 2. Results

### 2.1. Verification of the HFFD/LPS-Induced AD Model

**Figure 1:**
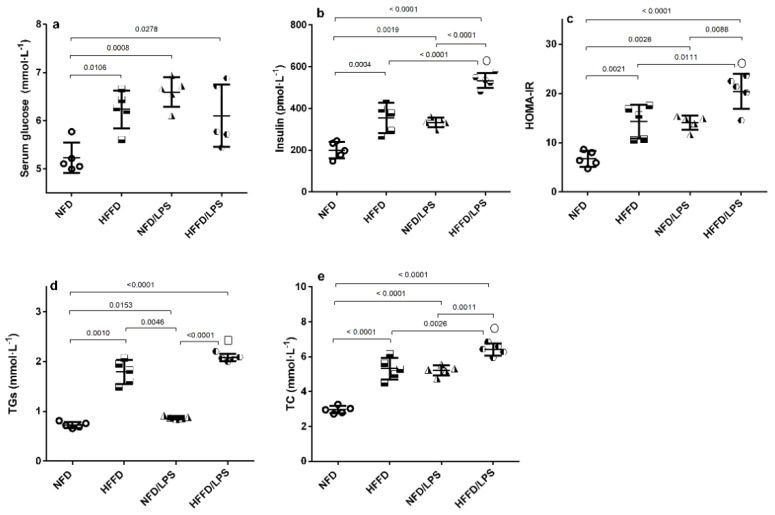
Effects of LPS and/or HFFD on glucose homeostasis parameters [(**a**) glucose, (**b**) insulin, (**c**) HOMA-IR], and lipid profile [(**d**) TGs and (**e**) TC] in rats after 9 weeks. Values are shown in scatter plots and expressed as mean ± SD (*n* = 5/group). Glucose, insulin, HOMA-IR and TC were analyzed using one-way ANOVA followed by Tukey’s post hoc test, while Welch’s ANOVA was performed followed by Dunnett’s T3 Multiple Comparison test for TGs to account for heterogeneity; *p* < 0.05. The symbols (□) and (○) indicate additive and synergistic interactions, respectively, using coefficient drug index (CDI). Rats were fed NFD or HFFD for 8 weeks and then received or not a single dose of LPS (2 mg·kg^−1^, i.p). All groups continued on NFD for another week. HFFD: high fat-fructose diet; HOMA-IR: Homeostasis Model Assessment of insulin resistance; LPS: lipopolysaccharide; NFD: normal fat diet; TC: total cholesterol; TGs: triglycerides.

Figure 1 shows that HFFD, LPS, and their combination caused a comparable significant (a) hyperglycemia compared to normal fat diet (NFD) rats. However, HFFD or LPS alone has elicited a state of IR indicated by (b) hyperinsulinemia and (c) homeostasis model assessment index of insulin resistance (HOMA-IR), values that were synergistically increased upon combining both HFFD and LPS, as compared to either insult alone using the coefficient drug index (CDI) test. Moreover, HFFD/LPS caused a synergistic upsurge in total cholesterol (TC) vs. either HFFD or LPS groups, while the elevation of triglycerides (TGs) was additive to surpass that of LPS only, to further support the augmented IR paradigm. 

**Figure 2:**
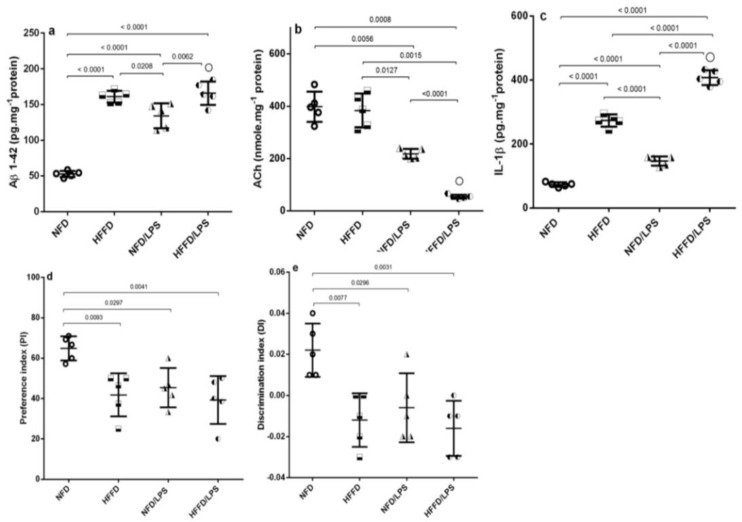
Effects of LPS and/or HFFD on hippocampal content of (**a**) Aβ1-42, (**b**) ACh, (**c**) IL-1β, and (**d**,**e**) PI/ DI during NOR test in rats after 9 weeks. Values are shown in scatter plots and expressed as mean ± SD (*n* = 5/group). All parameters were analyzed using one-way ANOVA followed by Tukey’s post hoc test, while Welch’s ANOVA was performed followed by Dunnett’s T3 Multiple Comparison test for ACh to account for heterogeneity; *p* < 0.05. (○) indicates synergistic interaction using coefficient drug index (CDI). Rats were fed NFD or HFFD for 8 weeks and then received or not a single dose of LPS (2 mg·kg^-1^, i.p). All groups continued NFD for another week. Aβ1-42: amyloid beta 1-42; ACh: acetylcholine; DI: discrimination index; IL-1β: interleukin 1 beta; NOR: novel object recognition; PI: preference index.

**Figure 3:**
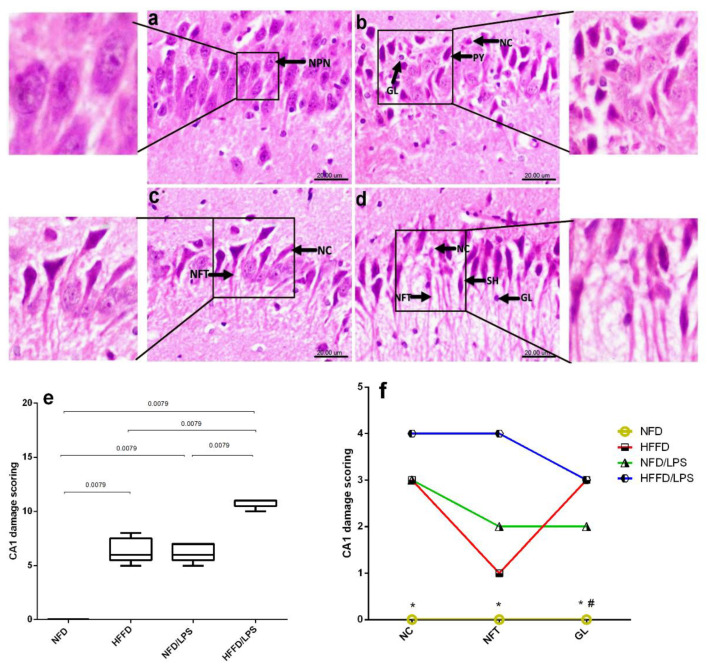
Effects of LPS and/or HFFD on hippocampal CA1 histopathological examination in rats after 9 weeks. The photomicrograph of H&E stained section from CA1 region of the hippocampus of (**a**) NFD shows normal histological architecture with tightly packed normal pyramidal neurons having large vesicular nuclei (NPN), while section of (**b**) HFFD, shows pyknosis (PY) and necrosis (NC) of pyramidal neurons with proliferation of glia cells (GL). Section of (**c**) NFD/LPS reveals necrosis (NC) of pyramidal neurons with neurofibrillary tangles (NFT) in some neurons and that of (**d**) HFFD/LPS, shows a marked necrosis (NC) of pyramidal neurons with flame-shaped neurofibrillary tangles (NFT) and proliferation of glia cells (GL) (Scale bar 20 µm, × 600). Panel (**e**) represents collective scores of CA1 damage presented in box and whisker and analyzed using the Mann–Whitney test between 2 groups, and panel (**f**) represents individual CA1 damage scores expressed as median (max-min) and analyzed using the Kruskal–Wallis test, followed by Dunn’s post hoc test (*p* < 0.05), as compared from (*) HFFD/LPS and (#) HFFD. Rats were fed with NFD or HFFD for 8 weeks; the NFD and HFFD groups either received a single dose of LPS (2 mg/kg^−1^, i.p) or not and all groups continued on NFD for another week.

In addition, the HFFD nutrition contributed to a state of low-grade inflammation that was synergistically enhanced by the administration of LPS, as depicted in Figure 2 as compared to NFD, HFFD, and LPS groups. The HFFD/LPS increased the hippocampal content of the surrogate AD marker (a) Aβ1-42 (3.2 fold) to override the LPS effect and boosted the content of the inflammatory cytokine (c) IL-1β (5.6 fold), effects that were associated by a sharp decrease in the hippocampal content of the neurotransmitter (b) ACh. These alterations indicated a synergistic worsening of the assessed parameters compared to the normal, as well as individual insults (LPS or HFFD). Besides these biochemical alterations, HFFD, LPS, or their combination has declined the animals’ preference to explore and discriminate the newly introduced object in the novel object recognition (NOR) test, to a comparable manner, when compared to NFD. These findings were further confirmed by the results of hippocampal CA1 microscopic examination (Figure 3); section of (a) NFD shows normal histological architecture with tightly packed normal pyramidal neurons having large vesicular nuclei, whereas section of (b) HFFD only shows pyknosis and necrosis of neurons, proliferation of glia cells and neurophagia of necrotic neurons. Sections from (c) LPS treated rats showed moderate neuropathic alterations as atrophy, shrunken and degeneration of some pyramidal neurons in the CA1 region associated with the appearance of NFTs in some neurons. Additionally, a severe neuropathic damage was recorded in the hippocampus CA1 region of rats treated with both (d) HFFD/LPS, where the examined section reveals marked degeneration and necrosis of the pyramidal neurons with flame-shaped NFTs, proliferation of glia cells, and neurophagia of necrotic neurons. The extent of the hippocampal CA1 histopathological damage is summarized in panels (e) and (f) to compare the collective and individual changes, respectively. Accordingly, the present results show the augmentation of neurotoxicity when LPS is injected into HFFD. 

### 2.2. Palonosetron, MLA, or Their Combination Improves Long-Term Spatial Working Memory in AD Rats

**Figure 4:**
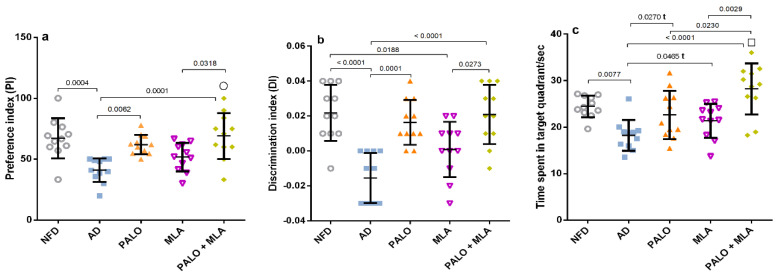
Effect of PALO and/or MLA on (**a**) PI and (**b**) DI during NOR test and (**c**) time spent in the target quadrant during MWM test in HFFD/LPS-induced AD model. Values are shown in scatter plots and expressed as mean ± SD (*n* = 11/group). Statistical analysis was performed using one-way ANOVA followed by Tukey’s post hoc test; (t) denotes significance using unpaired Student’s t-test between 2 groups; *p* < 0.05. The symbols (□) and (○) indicate additive and synergistic interactions, respectively, using coefficient drug index (CDI). PALO (0.1 mg·kg^−1^; i.p) and/or MLA (5.6 mg·kg^−1^; i.p) were administered 3 h after LPS injection and for 8 days. Training for MWM (day 4–7) was followed by probe test (on day 8). The NOR test habituation and familiarization phases (days 6 and 7) were followed by the probe test (day 8). MWM: Morris water maze.

After verification of the current AD model, the results in the main study revealed that the HFFD/LPS insult recapitulates the behavioral alteration in the NOR test (Figure 4), where these animals showed a decrease in both (a) preference index (PI) and (b) discrimination index (DI), as well as (c) a deterioration in the spatial working memory observed during the Morris Water Maze (MWM) task. On the contrary, palonosetron per se and its combination with MLA improved the rats’ memory during both tests; however, MLA alone amended only the rats’ spatial working memory during MWM test, as compared to AD group.

### 2.3. Palonosetron, MLA, or Their Combination Prevents the Hippocampal Microglial Depolarization in AD Rats

**Figure 5:**
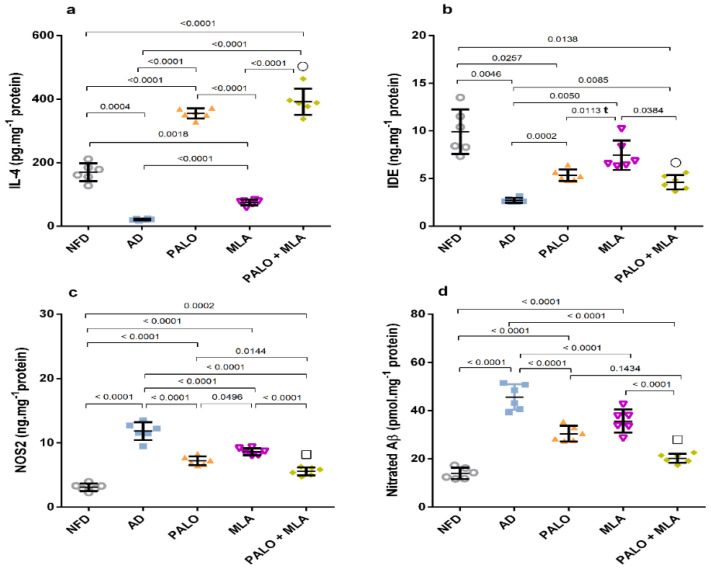
Effect of PALO and/or MLA on hippocampal contents of (**a**) IL-4, (**b**) IDE, (**c**) NOS2, and (**d**) nitrated Aβ in HFFD/LPS-induced AD model. Values are shown in scatter plots and expressed as mean ± SD (*n* = 6/group). NOS2 and nitrated Aβ were analyzed using one-way ANOVA followed by Tukey’s post hoc test, while Welch’s ANOVA was performed followed by Dunnett’s T3 Multiple Comparison test for IL-4 and IDE to account for heterogeneity; (t) denotes significance using unpaired Student’s *t*-test between 2 groups; *p* < 0.05. The symbols (□) and (○) indicate additive and synergistic interactions, respectively, using coefficient drug index (CDI). PALO (0.1 mg·kg^−1^; i.p) and/or MLA (5.6 mg·kg^−1^; i.p) were administered 3 h after LPS injection and for 8 days. IDE: insulin degrading enzyme; IL-4: interleukin 4; NOS2: nitric oxide synthase 2.

As presented in Figure 5, induction of the AD model diminished the hippocampal protective M2 microglia as evidenced by the (a) 88% decline in the anti-inflammatory cytokine IL-4 and the (b) insulin degrading enzyme (IDE). Conversely, the AD model activated the pro-inflammatory M1 phenotype, where it elevated (c) nitric oxide synthase 2 (NOS2) (3.8 fold) to enhance (d) nitration of Aβ (3.3 fold). Treatment with palonosetron successfully boosted IL-4, while MLA had a better effect on increasing IDE relative to other treatment regimens. However, their combination showed a synergistic increase in both parameters using the CDI test. Conversely, all treatments decreased NOS2 with the consequent reduction in the nitrated Aβ, with the combination regimen showing the best additive effect.

### 2.4. Palonosetron, MLA or Their Combination Suppresses the Hippocampal Canonical and Non-Canonical Activated Inflammasome Cascades in AD Rats

**Figure 6:**
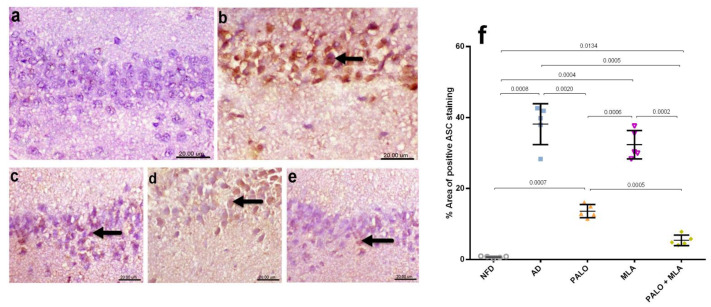
Effect of PALO and/or MLA on hippocampal CA1 immune-histopathological examination of ASC/TMS1 expression in HFFD/LPS-induced AD model. Immunohistochemical analysis of ASC in the CA1 region of hippocampus of rats reveals no ASC expression in the (**a**) NFD, while section of (**b**) AD group shows strong intracellular immune reaction presented by brown immunostaining expression (arrow). Section of (**c**) PALO treated group shows mild ASC expression (arrow), whereas section of (**d**) MLA treated group depicts moderate intracellular ASC expression (arrow). However, section from (**e**) PALO + MLA treated group shows a very weak ASC expression (arrow) (Scale bar 20 µm, ×600). Panel (**f**) represents the percent area expression from 5 randomly chosen fields in each section. Data were analyzed using one-way ANOVA followed by Tukey’s *post hoc* test; *p* < 0.05. PALO (0.1 mg·kg^−1^; i.p) and/or MLA (5.6 mg·kg^−1^; i.p) were administered 3 h after LPS injection and for 8 days.

**Figure 7:**
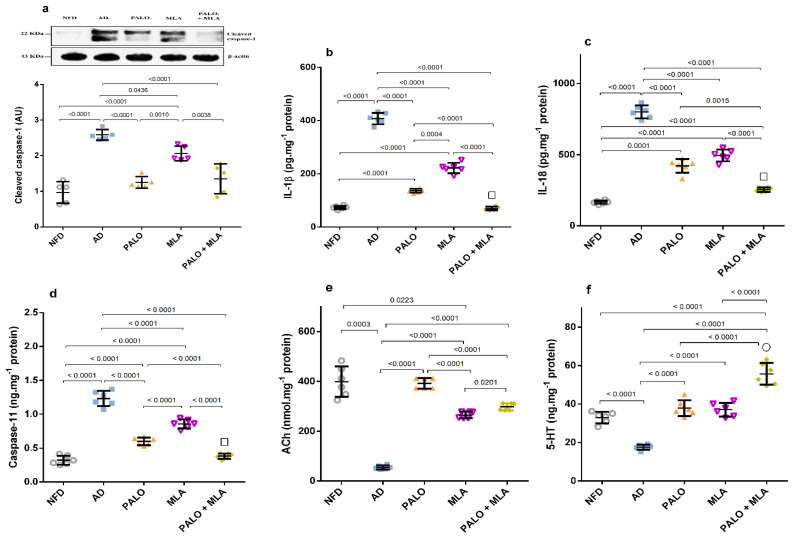
Effect of PALO and/or MLA on hippocampal contents of (**a**) cleaved caspase-1, (**b**) IL-1β, (**c**) IL-18, and (**d**) caspase-11, as well as (**e**) ACh and (**f**) 5-HT in HFFD/LPS-induced AD model. Values are shown in scatter plots and expressed as mean ± SD (*n* = 5–6/group). Panel (**a**) depicts representative cropped blot of cleaved caspase-1 and its densitometric analysis (*n* = 5/group). Cleaved caspase-1, caspase-11 and 5-HT were analyzed using one-way ANOVA followed by Tukey’s *post hoc* test (*n* = 6/group), while Welch’s ANOVA was performed followed by Dunnett’s T3 Multiple Comparison test for IL-1β, IL-18 and ACh to account for heterogeneity (*n* = 6/group); *p* < 0.05. The symbols (□) and (○) indicate additive and synergistic interactions, respectively, using coefficient drug index (CDI). PALO (0.1 mg·kg^−1^; i.p) and/or MLA (5.6 mg·kg^−1^; i.p) were administered 3 h after LPS injection and for 8 days. 5-HT: serotonin; ASC/TMS1: apoptosis-associated speck-like protein/the target of methylation-induced silencing-1; IL-18: interleukin 18.

In Figure 6, the AD model triggered inflammasome assembly, as indicated by the increased protein expression of ASC/TMS1, as compared to the NFD group. Figure 7 showed the activation of the inflammasome downstream molecules in rats with AD, depicted by the bolstered hippocampal content of (a) cleaved caspase-1 (2.6 fold), (b) IL-1β (5.6 fold), (c) IL-18 (4.9 fold), and the non-canonical marker (d) caspase-11 (3.8 fold), as compared to the normal group. However, among the two figures, treatment with palonosetron and its combination with MLA have normalized the protein expression of ASC and cleaved caspase-1, whereas the effect of MLA was confined to cleaved caspase-1, when compared to AD rats. In turn, the different treatment regimens succeeded to deter the downstream molecules in the ascending order of MLA, palonosetron, and their combination, which showed an additive effect. Conversely, the AD model almost depleted the hippocampal content of (e) ACh and halved that of (f) 5-HT, as compared to their normal counterpart. Nevertheless, ACh content was improved by MLA and the combination regimen, but restored by palonosetron. Regarding 5-HT, the single treatments have normalized it, but their combination increased it more to reach a synergistic level.

### 2.5. Palonosetron, MLA, or Their Combination Preserves the Hippocampal Architecture in AD Rats

**Figure 8:**
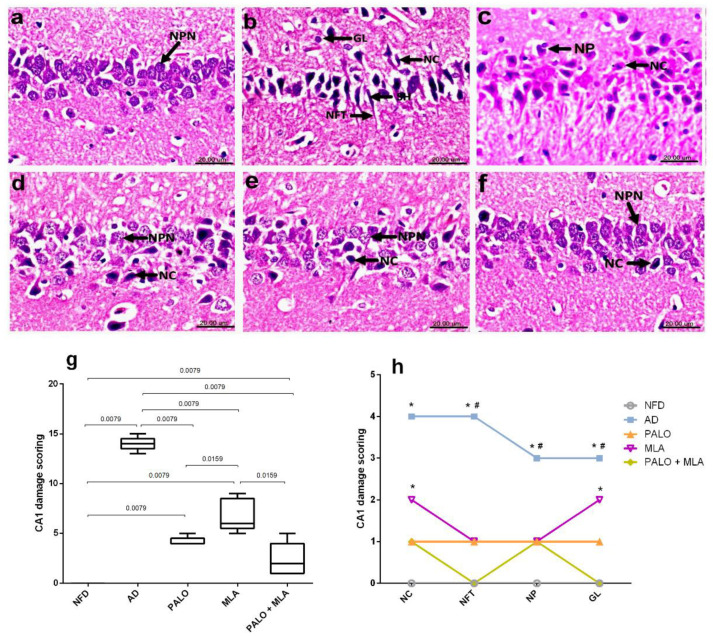
Effects of PALO and/or MLA on hippocampal CA1 histopathological examination in HFFD/LPS-induced AD model. Photomicrograph of H&E stained sections from CA1 region of hippocampus of rats in (**a**) NFD reveals the normal histological architecture of this area with tightly packed normal pyramidal neurons having large vesicular nuclei (NPN). Sections of (**b**,**c**) AD model shows marked shrunken (SH) and necrosis (NC) of pyramidal neurons, flame-shaped neurofibrillary tangles (NFT) proliferation of glia cells (GL) and neurophagia (NP). Sections of (**d**) PALO, as well as (**e**) MLA treated groups show necrosis (NC) of some neurons and other neurons appeared normal (NPN), while sections of (**f**) PALO + MLA treated group shows restored histologically normal neurons (NPN) with necrosis of sporadic neurons (NC) (Scale bar 20 µm, × 600). Panel (**g**) represents collective CA1 damage scores presented in box and whisker and analyzed using Mann–Whitney test to compare between 2 groups. Panel (**h**) depicts individual CA1 alteration scores expressed as median (max-min) and analyzed using the Kruskal–Wallis test, followed by Dunnett’s post hoc test (*p* < 0.05), as compared from (*) NFD and (#) PALO + MLA treated group. PALO (0.1 mg·kg^−1^; i.p) and/or MLA (5.6 mg·kg^−1^; i.p) were administered 3 h after LPS injection and for 8 days.

As presented in the microscopic photomicrographs (Figure 8), hippocampal CA1 section of (a) NFD rat reveals the normal morphology with tightly packed normal pyramidal neurons having large vesicular nuclei, whereas sections of (b and c) AD rats show severe neuropathic alterations in the CA1 area with marked shrunken, pyknosis and necrosis of pyramidal neurons with flame-shaped NFTs and proliferation of glia cells, as well as neurophagia. On the contrary, (d) a regression of the lesions is observed in section of palonosetron treated rats, where examined sections exhibited necrosis of some CA1 neurons, while others appeared normal. Moreover, (e) treatment with MLA has improved histopathologic changes and the hippocampus CA1 area shows necrosis of few neurons with an increased number of normal pyramidal neurons with a slight glia cells proliferation. Section from (f) the combined group, however, shows the restoration of the normal histological architecture of CA1 hippocampus area with necrosis of sporadic neurons in some sections. The extent of histopathological damage in the CA1 hippocampus area is summarized in panels (g) and (h), which represent the collective and individual changes, respectively. 

### 2.6. Palonosetron, MLA, or Their Combination Reduce Amyloid Plaques-Induced by HFFD/LPS in Rats

**Figure 9:**
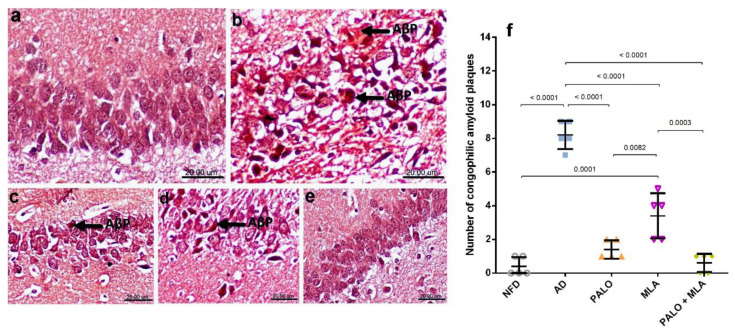
Effects of PALO and/or MLA on CA1 hippocampal histopathological examination of amyloid plaques in HFFD/LPS-induced AD model. Photomicrograph of Congo red stained sections from CA1 region of hippocampus of rats in (**a**) NFD shows the normal histology of neuronal cells with no deposition of amyloid β plaques (AβP). Section of (**b**) AD model shows multiple intracellular and extracellular deposition of AβP; meanwhile, sections of the (**c**) PALO treated group shows mild deposition of these plaques. Sections of the (**d**) MLA treated group shows multifocal moderate intracellular deposition of AβP, the section of (**e**) PALO + MLA shows no deposition of AβP (Scale bar 20μm, ×600). Panel (**f**) represents the number of congophilic AβP counted in 5 random non-overlapping microscopic field (×600) in each section. Data were analyzed using one-way ANOVA followed by Tukey’s post hoc test; *p* < 0.05. PALO (0.1 mg·kg^−1^; i.p) and/or MLA (5.6 mg·kg^−1^; i.p) were administered 3 h after LPS injection and for 8 days.

Congo red stained hippocampal CA1 tissue sections (Figure 9) of (a) normal control rats revealed normal histology and neuronal cells implying no deposition of Aβ plagues. On the contrary, CA1 hippocampal area of (b) AD rats exhibited multiple intracellular and extracellular deposition of Aβ plaques stained orange red in color. The Aβ depositions were reduced to moderate levels in (c) plaonosetron and (d) MLA treated groups, but were not detected in (e) the combined group. 

### 2.7. Palonosetron, MLA, or Their Combination Decrease Astrocytes Immunoreactivity in AD Rats 

**Figure 10:**
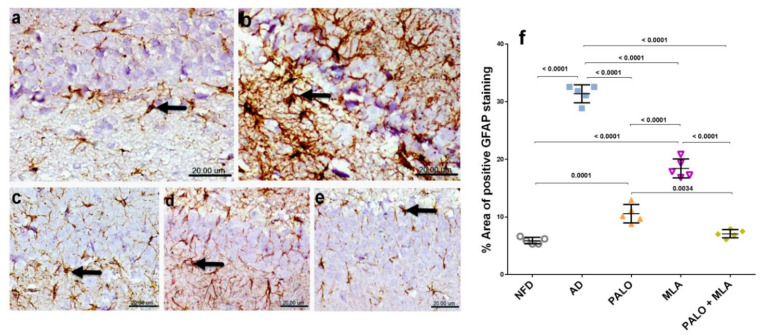
Effects of PALO and/or MLA on hippocampal CA1 immuno-histopathological examination of astrocytes in HFFD/LPS-induced AD model. Immunohistochemical analysis of GFAP expression in the CA1 region of hippocampus of rats in (**a**) NFD group shows normal small-sized astrocytes with lightly stained GFAP positive short processes (arrow), while sections of (**b**) AD model show strong immunoreactivity of hypertrophied astrocytes with deeply stained GFAP positive brown processes (arrow). On the other hand, section of (**c**) PALO treated group shows mild GFAP positive expression (arrow) and section of (**d**) MLA treated group shows moderate GFAP expression (arrow). Meanwhile, section of (**e**) PLAO + MLA treated group depicts normal small-sized astrocytes with lightly stained GFAP (arrow) processes (Scale bar 20 μm, × 600). Panel (**f**) represents the % area of positive GFAP staining from 5 randomly chosen fields in each section. Data were analyzed using one-way ANOVA followed by Tukey’s *post hoc* test; *p* < 0.05 PALO (0.1 mg·kg^−1^; i.p) and/or MLA (5.6 mg·kg^−1^; i.p) were administered 3 h after LPS injection and for 8 days. GFAP: glial fibrillary acidic protein.

As depicted in Figure 10, microscopic examination of CA1 hippocampal area section of (a) normal control rats revealed normal small-sized astrocytes with lightly stained glial fibrillary acidic protein (GFAP) positive short processes. Conversely, strong immunoreactivity of hypertrophied astrocytes with deeply stained GFAP positive brown processes was detected in the hippocampus CA1 area of (b) AD rats. Rats treated with (c) palonosetron exhibited mild immune-reactive astrocytes with lightly stained processes, whereas (d) MLA treated rats showed moderate immune-reactivity and (e) the combined group revealed normal small-sized astrocytes with lightly stained GFAP positive short processes.

## 3. Discussion

In a novel HFFD/LPS AD-like model, palonosetron and, unexpectedly, MLA have alleviated cognitive dysfunction, improved memory, and recovered histological architecture. Besides increasing the hippocampal contents of 5-HT3 and ACh, the tested agents skewed M1 microglia to the protective M2 phenotype confirmed by the increased IL-4 and the decreased NOS2-mediated Aβ nitration, besides activating IDE to facilitate the clearance of Aβ and to halt AD progression. Additionally, treatment regimens deterred inflammasome assembly and activation to enhance cell survival by decreasing pyroptosis. It is worth mentioning that the effect of palonosetron was mostly superior to MLA to corroborate a better cognitive improvement and histopathological scoring. 

One factor that perturbs the permeability of the BBB is the alterations in the gut microbiota induced by the consumption of HFD [19]. These microorganisms are essential for extracting nutrients, as well as the production of vital byproducts implicated in various processes, among which is the adequate central nervous system (CNS) functioning [32]. Therefore, factors that disturb the gut microbiome equilibrium can alter the availability of valuable metabolic precursors and consequently affect brain development [33], besides increasing the gut permeability [17]. The latter ends up with a leaky gut that facilitates the transfer of gram-negative bacteria-derived LPS into the blood to promote endotoxemia and systemic inflammation that deteriorates BBB fence function [19]. Accordingly, our notion on establishing the current model was based on the fact that HFFD alters gastrointestinal tract (GIT) microbiota and increases both intestinal [17] and BBB [19] permeability and that the bacterial endotoxin LPS co-localizes with amyloid plaques in AD human brains [34]. Moreover, the two insults, LPS [35] and HFFD [13,14,35] are able to initiate central inflammation and impair cognition. Hence, our model (HFFD/LPS) was induced to replicate sporadic AD in humans and the HFFD was also used to ease the central access of LPS, which poorly crosses BBB [36].

In the pilot study, the model was verified by the impaired cognition and the excessive formation of hippocampal Aβ1-42 and IL-1β along with the sharp decline in the neurotransmitter ACh, effects that surpassed those mediated by either insult alone. The behavioral and biochemical results were further supported by the histopathological examinations. Interestingly, in the pilot experiment, we noticed that the HFFD alone has elevated soluble Aβ without decreasing ACh to suggest that the contribution of Aβ alone in the degeneration of cholinergic neuron is still questionable. In this regard, controversial results were reported; some findings support ours, in which westernized HFD and fructose administration adversely affected working and spatial memory in rats without a significant change in brain ACh level, but they altered the expression of Aβ metabolism-associated molecules that are responsible for Aβ deposition [37,38]. On the contrary, a nexus between accumulated Aβ and cholinergic degeneration was reported [39]. The latter picture, however, was noticed in the current AD model upon the combination of HFFD and LPS, where Aβ1-42 was significantly elevated and the hippocampal level of ACh was depleted, changes that were accompanied by a further damage in the hippocampal structure.

After its verification, the present model was used to assess the anti-neuropathic potential of palonosetron, being a 5-HT3 blocker. Though neuroprotection was previously ascribed to tropisetron, another 5-HT3 receptor blocker in Aβ-challenged rat cortical neurons [40], however, the in vivo anti-dementia effect of palonosetron is first reported here, where the drug succeeded to improve cognition during both NOR and MWM tests. Although we used MLA, alone and in combination with palonosetron to investigate the possible involvement of α7nAChR in palonosetron’s effects, surprisingly, MLA per se showed neuroprotection and has unexpectedly intensified the beneficial outcomes of palonosetron. Our findings do concur with earlier studies, since the previous reports about the MLA effects appeared to be controversial. While in one study the blockage of α7nAChR in normal rats by MLA has induced an in vivo model of cognitive impairment [28], another one showed that α7nAChR receptor blockade in primary neuron-enriched cultures afforded neuroprotection [41]. Moreover, the blocker MLA was reported to inhibit methamphetamine-induced production of reactive oxygen species in mouse striatum [42] and to protect against LPS-mediated release of tumor necrosis factor (TNF)-α from microglia [43]. It also alleviated Aβ-induced cytotoxicity [44] via inhibiting autophagy with the involvement of mammalian target of the rapamycin (mTOR) pathway in SH-SY5Y cells. Moreover, the protective effect of MLA was further confirmed by the results of the MWM test to consolidate the findings of previous in vitro/in vivo studies [45,46]. Indeed, the concomitant blockage of both receptors herein elicited a better outcome on both cognition and memory, results that were mirrored in the morphological picture of the hippocampus. 

Apart from the enhancements of function and structure, the current treatments, especially the combined regimen, have increased the protective M2 phenotype over the pathological one (M1), which represent the central innate immune cells responsible in large part for neuroinflammation in AD. Based on the phenotype activated, microglia can produce either cytotoxic or neuroprotective effects. NOS2 and the consequent nitration of Aβ are indicators for M1 activation [47], while IL-4 and IDE are considered markers for activated M2 microglia [47]. In pathological conditions, such IR [48] and AD [47], the immune responses are usually skewed to the M1 phenotype, which is greatly accompanied with cell loss or cell dysfunction. In AD, microglia are activated towards the M1 phenotype when NLRP3 recognizes Aβ aggregates and opens up to form the inflammasome. In turn, activated microglia, especially those surrounding Aβ plaques, secretes and releases IL-1β [49], thus presenting another marker for activation and accentuate the role of NLRP3 inflammasome in this process, a verity supported by the failure of NLRP3-/- mice to respond to Aβ and to produce IL-1β [49].

Indeed, Aβ phagocytosis [49], HFD [50] and LPS [51] activate microglia to trigger chronic neuroinflammation resulting in cognitive deficits [52]. These facts concur with the current findings, where the HFFD/LPS model activated the pro-inflammatory M1 microglia over M2, as confirmed by the boosted NOS2 and the increased nitro-Aβ along with the sharp decline in IL-4 and the IDE. Previous studies have highlighted the crucial role of IL-4 in abating AD as reported in a sporadic AD case-control study [53] by inducing the protective M2 phenotype, besides the IDE-dependent Aβ clearance [54], which in turn attenuates Aβ-induced synaptic plasticity/cognition impairments [55,56]. Moreover, the increased NOS2-induced Aβ nitration was reported to aggravate Aβ seeding and plaque formation to expand the AD pathology [57]. Hence, the aptitude of the current treatments to augment the M2 microglia markers (IL-4, IDE) and to reduce the M1 related parameters (NOS2, nitrated Aβ) signify in part the improved behavior and structure of the treated groups. Moreover, the effect of MLA to initiate M2 microglia can be linked to the blockade of α7nAChRs along with the inactivation of NOS2 and the deterred Aβ. It was previously reported that Aβ assemblies can bind to α7nAChRs in neurons to disrupt synaptic function in AD [26] by converting the “silent” glutamatergic synapses into functional ones [58].

The second axis upon which the tested agents rely to protect neurons is the inhibition of inflammasome assembly and activation. Inflammasome, a large multiprotein complex, is activated in response to cytokines produced from activated microglia in response to Aβ phagocytosis [49]. Despite inflammasome helping the cell to tackle tissue damaging stimuli, its abandoned activation contributes in the emergence of several diseases, including the neurodegenerative ones. Notably, the NLRP3 inflammasome is the most extensively studied type, with its activation involving two steps, priming (signal 1) and activation (signal 2). The signal 1 is mediated via the sensing of TLR4 to extracellular stimuli, which in turn activates NF-κB to increase the formation of pro-IL-1β, as well as the transcription of the NLRP3 protein. Signal 2, on the contrary, is initiated by pathogen associated molecular patterns (PAMPs) and danger associated molecular patterns (DAMPs) promoting NLRP3 inflammasome assembly and the consequent caspase-1- mediated activation and secretion of IL-1β, IL-18 and gasdermin D (GSDMD). The latter forms pores in the plasma membrane, causing the escape of the cytoplasmic signaling molecules outside the cell, as well as an inflammatory form of cell death termed pyroptosis [7]. Our study further confirms the crosstalk between the activated microglia and inflammasome that was reported recently [59], where the LPS [5] and HFD [60] insults represent DAMPs that activate the microglia TLR4 to trigger inflammasome priming and assembly [59]. In our model, activated inflammasome was reinforced by the increased protein expression/contents of ASC/TMS1 and the cleaved caspase-1 to indicate the assembly of inflammasome moiety and its activation. In turn, the active caspase-1 promotes the maturation of the inflammatory cytokines IL-1β and IL-18 from their proforms, as evidenced here and hitherto [49]. These events, however, augment the neuroinflammatory environment and aggravate AD progression, where the latter cytokines and their upstream molecules are further activated by the phagocytosis of the fibrillary Aβ by microglia [49] to be accrued in lysosomes. This upshot leads eventually to the rupture of lysosomes with the release of their components to reactivate NLRP3 with the formation of ASC specks and the maturation of their downstream cytokines in a feed forward series. Moreover, and in a vicious cycle, the increased ASC specks, released from pyroptotic cells, advance also the formation of Aβ plaque to be engulfed again by microglia to recirculate the previous cascades [61]. These facts were recapitulated in our novel model to be responsible for the neuroinflammatory environment and the impaired cognition. However, palonosetron alone or with MLA has corrected this whole picture as the tested treatments were able to decrease the canonical NLRP3 inflammasome, in which the ASC/TMS1 moiety and its downstream molecules were curtailed, as documented by the immunohistochemical examination, to confer neuroprotection as evidenced previously [47,62] and to drift microglia towards the anti-inflammatory M2 type along with mobbing up Aβ [47,61].Furthermore, by enhancing the anti-inflammatory cytokine IL-4, these agents were able to intersect the tethering between microglia, inflammasome, and Aβ, where the M2 microglia marker IL-4 was proven to hinder the inflammasome cascade and IL-1β, as well as Aβ [55,63].

Nonetheless, blocking α7nAChR also inhibited inflammasome, but in a non-canonical pattern; MLA decreased caspase-1 and IL-1β/IL-18 maturation, but not ASC/TMS1, an essential molecule in the NLRP3 inflammasome assembly. Indeed, in a non-canonical manner, the NLRP3 inflammasome could be activated by caspase-11, which represents another stimuli for triggering caspase-1 [64], without the involvement of ASC/TMS1 [65]. This fact, therefore, can explain the ability of MLA to suppress inflammasome cascade by inhibiting caspase-1/IL-1β/IL-18 molecules following the inactivation of caspase-11, as reported herein. The currently activated caspase-11 could be linked to the presence of cytosolic LPS that acts as caspase-11 agonist [66] and/or the HFFD [67]. Hence, the inactivation of caspase-11 by palonosetron and/or MLA gives a further support for the inflammasome disassembly/deactivation, besides the suppression of ASC/TMS1 expression by palonosetron.

Since the role of both IL-1β [68,69] and IL-18 [70] is well established in AD, their significant increase ensues that the inflammatory story does not end by their activation. However, in a feed forward cycle, these two inflammatory cytokines; viz., IL-1β [71] and IL-18 [70], activate NLRP3, ASC/TMS1, and caspase-1 [71] by acting on their receptors; moreover, they increase the amyloid precursor protein [72,73] and beta-site APP-cleaving enzyme-1, which is responsible for the aberrant Aβ1-42 cleavage [73] to additionally clarify the HFFD/LPS/-mediated microglia/inflammasome activation. Thus, palonosetron and/or MLA via the inhibition of caspase-11 and -1, as well as IL-1β and IL-18 intervene with the non-stop series of cytokine/NLRP3 inflammasome/microglial activation to reduce AD neuroinflammation.

Additionally, the anti-dementia/neuropathy effect of the tested compounds is partly mediated by increasing neuronal survival to present the third trail through which our blockers acted to pin down their beneficial effect. In our study, the histopathological results documented the apoptotic and necrotic pyramidal neurons cell death, whereas the biochemical parameters indicated the partake of pyroptosis in cell demise. This type of cell death is a highly controlled inflammatory programmed cell death that depends on the activation of caspase-1 and -11 along with the inflammatory downstream cytokines IL-1β and IL-18 [74]. Indeed, Xie & Zhao [75] have reviewed the role of pyroptosis in several neurological diseases suggesting its crucial role in AD, events that support the current HFFD/LPS-induced neurodegeneration. Accordingly, the interplay of the different types of cell death, evidenced in the present work, could be one reason behind the compromised 5-HT and ACh levels in the AD model, whereas the palonosetron and/or MLA-mediated neuronal survival further highlights their neuroprotective capacity and the retrieval of both 5-HT and ACh, neurotransmitters that are partially responsible for the observed memory improvement. 

## 4. Materials and Methods

### 4.1. Animals

Male Wistar rats aged 6–8 weeks old (90–100 g) were obtained from the Animal Production Research Institute (Giza, Egypt) and were left for a week to accommodate in standard polypropylene cages at the animal facility of the Faculty of Pharmacy, Cairo University (Cairo, Egypt). Animals were maintained under constant environmental conditions of 12/12 h dark/light cycles and a temperature of 25 ± 2 ℃ and were fed standard rat pellet diet and water *ad libitum* prior to dietary manipulation. The study followed the recommendations in the Guide for the Care and Use of Laboratory Animals of the National Institutes of Health [76] and adhered to ARRIVE guidelines. The protocol was approved by the Research Ethics Committee of the Faculty of Pharmacy, Cairo University (Permit Number: PT 2128). The animals were distributed among the experimental groups using a completely randomized design, ensuring the weight of rats in all groups was comparable and all tests were performed by operators blinded to the groups. At the end of the experiment, rats were euthanized by thiopental, and blood samples were collected from the jugular vein.

### 4.2. Drug and Chemicals

LPS (O55:B5), palonosetron hydrochloride (Emegrand), and MLA citrate were purchased from Sigma-Aldrich (St. Louis, MO, USA), Grand Pharma for Pharmaceutical Industries (Cairo, Egypt), and Tocris Bioscience (Bristol, UK), respectively. Moreover, the diet related components, *viz.*, cholesterol and fructose (Uni Fructose) were procured from Panreac (Barcelona, Spain) and UNIPHARMA (Cairo, Egypt), respectively, whereas sheep fat was obtained from a commercial source. Any other chemical used was of analytical grade. 

### 4.3. Development of HFFD/LPS Neuroinflammation Model 

This model of AD in rats was designed to resemble partly the hippocampal changes and potential mechanisms involved in sporadic AD in humans; *namely*, Aβ deposition, neuroinflammation, degeneration of cholinergic neurons, IR state, lipid metabolism abnormalities, and gut microbiota disorders, as recently reviewed [77]. For the induction of this model, a pilot study was conducted to verify the potential impact of injecting LPS to HFFD fed rats on intensifying neuroinflammation that associate AD-like models. Briefly, 20 rats were randomly and evenly divided into two main dietary groups (*n* = 10 each); rats in the first group received NFD [≈3000 kcal·g^−1^: fat as oils (3%), protein (21%), carbohydrate as starch (60%), fibers (3%), and vitamins and minerals (3%)]. However, animals in the second group were fed HFFD [≈5300 kcal·g^−1^] composed of oils (3%), sheep tail fat (15%) cholesterol powder (1%), protein (21%), carbohydrate as starch (60%), fibers (3%), and vitamins and minerals (3%) with fructose (20%) in drinking water [78]. After 8 weeks, the development of an IR state in HFFD rats was confirmed by the HOMA-IR [79], calculated from the serum measurements of 10 h fasting glucose and insulin levels, as well as the elevated serum levels of TGs and TC (Data not shown). Afterwards, animals in the two main groups were subdivided into two subgroups (*n* = 5 each) to be classified into NFD (saline; group I), HFFD receiving saline (group II), NFD/LPS (2 mg·kg^−1^ in saline, i.p) [35] designated as group III, and HFFD/LPS (group IV). Animals in all groups were left for 8 days on NFD. These groups are used to compare between the impact of the different insults alone (LPS or HFFD) and their combination. 

The impact of HFFD, LPS or their combination on memory was assessed using the NOR test, which examines the ability of rats to recognize a novel object based on the animal’s natural preference. The test started 3 days before euthanasia and each phase (*viz.,* habituation, familiarization, and long-term memory) was carried out on a separate day. At the end of the behavioral test, rats were anaesthetized using a high dose of thiopental (100 mg·kg^−1^, i.p) and blood was rapidly withdrawn from the jugular vein and the sera were used to determine markers of IR (glucose, insulin to calculate HOMA-IR) and lipid profile (TGs, TC). Afterwards, one hemisphere was kept in 10% neutral buffered formalin for the histopathological examination, whereas the hippocampus was isolated on ice from the other hemisphere and homogenized in phosphate buffer saline (PBS) to measure the contents of selected biomarkers, *namely*, Aβ1-42, IL-1β, and ACh.

### 4.4. Experimental Design of the Main Study

**Figure 11:**
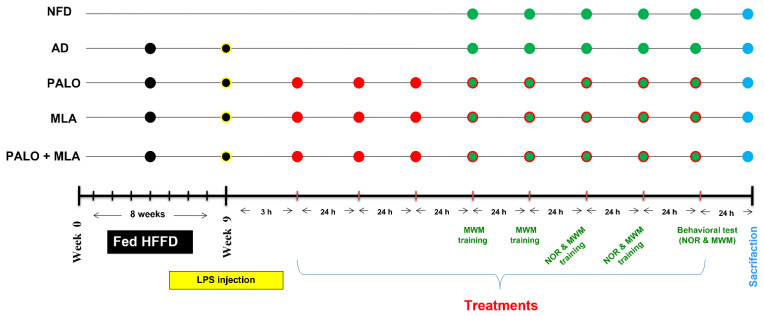
Experimental design time line. For 8 weeks, rats were fed NFD or HFFD to be injected on the last day with a single dose of saline or LPS (2 mg·kg^−1^, i.p), respectively. The HFFD/LPS rats were left untreated (AD model) or injected with PALO (0.1 mg·kg^−1^; i.p) and/or MLA (5.6 mg·kg^−1^; i.p) for 8 days starting 3 h after LPS injection. In the combined group, MLA was injected 15 min before PALO. On day 4 after LPS, MWM training was performed and the probe test was done on day 8. The NOR test, habituation and familiarization phases (days 6 and 7) were followed by the probe test (day 8). Rats were euthanized 24 h after the last dose of treatment.

After verification of the model and that the combination regimen has superseded that of either insult alone, the two insults were used to induce the AD model, which was used in the main part to study the pharmacological effects of the tested agents and their effect on the assessed parameters. For the main experimental study, another 55 male Wistar rats were initially divided into the same two dietary regimens; *viz.*, NFD and HFFD. Eleven rats were fed NFD and received saline (vehicle) to serve as the normal control group, whereas the remaining 44 rats were fed in-house prepared HFFD for 8 weeks then injected once with LPS after IR was confirmed and were referred to as AD rats; the changes in body weight were recorded throughout the experiment period. The HFFD/LPS rats were randomly divided into four subgroups (*n* = 11 each) to be either left untreated (AD control) or treated with palonosetron (0.1 mg·kg^−1^, i.p), MLA (5.6 mg·kg^−1^, i.p; [80]), or their combination. Treatments (palonosetron or MLA) were dissolved in saline and were injected starting 3 h after the LPS injection, whereas in the combined regimen, MLA was injected 15 min before each palonosetron dose. Injections continued for 7 consecutive days with a total of 8 injections during which all animals were fed NFD. The NOR long-term memory and MWM probe tests were performed on the last day of treatments (day 8). Figure 11 summarizes the experimental design.

### 4.5. Behavioral Tests

#### 4.5.1. NOR Test

The test started on day 6, where in the habituation phase each rat was allowed to freely explore the area in the absence of objects for 120 s, while on the second day and during the familiarization phase, each rat was placed in the same area, but in the presence of two identical sample objects (A + A) for 120 s. The different shape objects (cube and cylinder) to be discriminated were 7 cm high made of wood and could not be moved by the rat. To prevent any bias to explore the objects, the rat was released against the center of the opposite wall with its back facing the objects. In the long-term memory test phase (day 8), the rat was placed in the area with 2 different objects, a familiar one and a novel one (A + B, respectively) and the animal was left to explore the objects for 120 s. In this test, the time spent in exploring each object was recorded [81] in addition to the calculation of the preference (PI) and discrimination (DI) indices according to previous literature [82,83].

#### 4.5.2. MWM Test

The test is used to evaluate the spatial learning and memory that are responsive to hippocampal damage [84]. Briefly, a large circular pool of 150 cm diameter and 50 cm depth was filled with tap water of around 26 °C. The water maze is divided into quadrants with 4 starting positions, North (N), South (S), East (E), and West (W). The N position was used to place a platform (15 × 15 cm) exposed 2.5 cm above the water surface to be recognized by the animal. The whole test was performed over 5 days and training started on day 4 following LPS injection to the HFFD group or an equivalent time point for the NFD group. On the first 4 days of the test, animals in all groups were trained to find the platform, where each animal underwent 3 daily trials from 3 different platform locations for 4 consecutive days without repeating the trial sequence on 2 successive days (SEW, ESW, WSE, SWE, etc.). The animal was gently lowered into the water down facing the pool wall and allowed to swim/search for the platform for a maximum of 120 s. Rats that reached the platform were left for 10 s before being removed; however, those which did not find the platform in 120 s were guided by hand to the platform location and were left there for 30 s. The escape latency for each rat was daily recorded in the acquisition trials; however, for the probe trial the platform was removed on the 5th day of the test. Each animal was tested by being placed in quadrant (S), the farthest to the platform, left to swim for 120 s and the time spent in the platform quadrant (N) was recorded. Notably, animals tested first were chosen by rotation on the different groups, where one rat from each group was chosen randomly to be tested and then a second rat and so on for the rest of the animals. On day 8 of treatment, all animals performed NOR test (which requires a minimum effort for object exploration) before the MWM to avoid the exhaustion of the animals that may affect their performance.

### 4.6. Biochemical Analysis

One day after the behavioral tests, the brains were dissected out and the intact hemispheres were harvested on ice after euthanasia. The hippocampi of 6 rats/group were homogenized in PBS for ELISA estimations of IDE, IL-4, NOS2, caspase-11, IL-1β, IL-18, 5-HT, and ACh. Estimation of hippocampal nitrated Aβ content was performed using a previously published [57] ELISA procedure. Data points in each independent experiment are generated from the average of 2 replications. In the other 5 rats/group, the hippocampus of one hemisphere was kept in RIPA buffer for the estimation of cleaved caspase-1 by Western blot analysis, while the other hemisphere was immediately fixed in 10% neutral buffered formalin for histopathological and immunohistochemical examinations. All methods were carried out in accordance with the relevant guidelines and regulations.

#### 4.6.1. Assessment of Serum Insulin, Glucose, and Lipid Profile 

The ELISA technique was carried out for the assessment of serum insulin (RayBiotech, Peachtree Corners, GA, USA; Cat# ELR-Insulin) and the SPINREACT Kits (Girona, Spain) were used for the colorimetrical estimation of glucose (Cat# MD41011), TGs (Cat# MD41031), and TC (Cat# MD41021). All procedures were carried out following the manufacturers’ instructions. 

#### 4.6.2. Assessment of Hippocampal Aβ1-42, Nitrated Aβ, NOS2, IL-4, and IDE

The following biomarkers were assessed using the corresponding ELISA kits obtained from LSBio (Seattle, WA, USA) for Aβ1-42 (Cat# LS-F23254), Elabscience (Houston, TX, USA) for IDE (Cat# E-EL-R2455) and MY BioSource (San Diego, CA, USA) for both NOS2 (Cat# MBS2702569) and IL-4 (Cat# MBS355442). The 3NTyr10-Ab (Merck, MA, USA; Cat# MABN779) was used for the assessment of nitrated Aβ [57] by ELISA. The parameters were assessed according to the manufacturers’ transcripts.

#### 4.6.3. Assessment of Hippocampal ACh and 5-HT

The two neurotransmitters ACh (MY BioSource, San Diego, CA, USA; Cat# MBS043949) and 5-HT (CAT# MBS166089) were assessed using MYBioSource ELISA kits according to the manufacturer’s instructions.

#### 4.6.4. Determination of Hippocampal Inflammasome Biomarkers

Caspase-11 (MYBioSource, San Diego, CA, USA; Cat# MBS008490), IL-1β (RayBiotech, Peachtree Corners, GA, USA; Cat# ELR-IL-1b), and IL-18 (Abcam, Waltham, MA, USA; Cat# ab213909) were measured using ELISA kits according to the manufacturers’ protocols. In the meantime, hippocampal content of cleaved caspase-1 was determined by Western blot technique, where the respective protein was extracted and separated by electrophoresis using SDS-PAGE. The gel was assembled in a transfer sandwich using PVDF membrane that was placed in the transfer buffer (25 MmTris, 190 mM glycine, and 20% methanol). The system was run for 7 min at 25 V to allow for the transfer of protein bands from gel to membrane using Trans-Blot Turbo (BioRad, Hercules, CA, USA) followed by blocking the membrane in Tris-buffered saline with Tween 20 (TBST) and 3% bovine serum albumin (BSA) at room temperature for 1 h. Cleaved caspase-1 (Cell Signaling Technology, Danvers, MA, USA; Cat# 4199, RRID: AB_1903914) and β-actin (anti-rabbit; Novus Biologicals, Littleton, CO, USA; Cat# NB100-56874, RRID: AB_837512) primary antibodies were diluted 1:500 in TBST and incubated overnight at 4 °C. After rinsing with TBST, the membrane was incubated with the HRP-conjugated secondary antibody (1:500; goat anti-rabbit; Novus Biologicals; Cat# NB120-6023, RRID: AB_790436) for 1 h at room temperature. After a second rinse with TBST the chemiluminescent substrate (ClarityTM Western ECL substrate; BioRad; Cat#170-5060) was applied following the manufacture’s protocol and the signals were captured using a CCD camera-based imager. Finally, the band intensities were normalized to β-actin using the ChemiDoc MP imager (BioRad).

### 4.7. Histopathological and Immunohistochemical Examinations

After fixation in formalin, the brain specimens were dehydrated in alcohol, cleared in xylene and embedded in paraffin wax. Paraffin blocks were sectioned at 4–5 μm and stained with Hematoxylin and Eosin (H&E) for histopathological examination and Congo red for the demonstration of amyloid plaques by a light microscope (Olympus BX50, Tokyo, Japan) under a high power magnification (×600) [85]. The histopathological alterations in the CA1 region of the hippocampus, including degeneration and necrosis of pyramidal cells (NC), neurofibrillary tangles (NFT), neurophagia (NP), or gliosis (GL) were graded by an experienced pathologist blinded to the groups from 0 to 4. Score (0) indicates no changes, score (1) indicates <10% changes, score (2) indicates 20–30% changes, score (3) indicates 40–60% changes, and score (4) indicates >60% changes with a total score of 12 in pilot groups (NC, NFT and GL) or 16 in treatment groups (NC, NFT, NP and GL) [86]. Each value was calculated from 5 randomly chosen fields in each section. Meanwhile, congophilic amyloid plaques were counted in five random non-overlapping microscopic field (×600) in Congo red stained sections and the data obtained were statistically analyzed.

The paraffin blocks were also sectioned from the different groups and immuno-stained with polyclonal Anti- ASC/TMS1 (Biospes, Chongqing, PRC; Cat#YPA1696, RRID: AB_2832254) primary antibody (1:200) containing 0.01 M TBS (pH 7.4) with 1% BSA, 0.03% proclin 300 and 50% glycerol. Quantification of ASC was estimated by measuring the area % expression from 5 randomly chosen fields in each section and averaged using image analysis software (Image J, version 1.46a, NIH, Bethesda, MD, USA).

#### Glial Fibrillary Acidic Protein (GFAP)

The deparaffinized and rehydrated sections were incubated with GFAP monoclonal antibody (1:500; Dako, N-series Ready to use primary antibody, Santa Clara, CA, USA). The immunostaining was amplified and completed by Horseradish Peroxidase complex (Dako, REALTM EnVision TM/HRP, Mouse ENV). Sections were developed and visualized using 3,3-diaminobenzidine (Dako, REALTM DAB + Chromogen). The substrate system produced a crisp brown end product at the site of the target antigen. Sections were counterstained with haematoxylin, then dehydrated in alcohol, cleared in xylene and cover slipped for microscopical examination. Quantification of GFAP was estimated by measuring the area % expression from 5 randomly chosen fields in each section and averaged using image analysis software (Image J, version 1.46a, NIH, Bethesda, MD, USA) by a blinded observer.

### 4.8. Calculation of Combination Effect Using Coefficient Drug Index (CDI)

The CDI [87] analyzes the interaction between the combined and single groups using the following formula: CDI = AB/(A × B). In the pilot experiment, AB is the ratio of the combination insult group (HFFD + LPS) to normal control group (NFD) and A or B is the ratio of the HFFD or NFD/LPS group to the normal control group (NFD). In the main experimental study, however, AB is the ratio of the combination treatment group (PALO + MLA) to model control group (AD) and A or B is the ratio of the PALO or MLA group to model control group (AD). Thus, the respective synergistic, additive, or antagonistic interaction is indicated as CDI < 1, =1, or >1.

### 4.9. Data and Statistical Analysis

Estimation of group size was performed using G*Power software version 3.1.9.7 (Heinrich-Heine-University, Düsseldorf, Germany) with a power of 0.80 and an alpha of 0.05 for comparison of four (pilot study) or five (main study) groups and effect size estimated was 0.9, so sample size estimation was *n* = 5/group. All data are shown in scatterplots and expressed as mean ± SD. The number of animals in each group was equal by design (*n* ≥ 5) and refers to every individual rat used in the respective experiment. All data were analyzed without any transformations and tested for heteroscedasticity using Bartlett’s test. Differences between groups of homogenate variances were tested for significance using unpaired *t*-test (two groups) or ordinary analysis of variance (ANOVA) followed by Tukey’s Multiple Comparison test as the post hoc test (more than two groups). However, Welch’s ANOVA was performed followed by Dunnett’s T3 Multiple Comparison test with individual variances computed for each comparison to account for heterogeneity; all tests were two-tailed. The non-parametric data (scores) were analyzed using Kruskal–Wallis (non-parametric ANOVA) test, followed by Dunn’s post hoc test to compare between the different groups and the Mann–Whitney test was used to compare scores between two groups only. The GraphPad prism software, version 8 (GraphPad Software, San Diego, CA, USA) was used to analyze the data and to draw the attached figures. The level of statistical significance was accepted at *p* < 0.05.

## 5. Conclusions

In conclusion, the current study denotes that exposing HFFD rats to LPS augments AD-associated molecular, functional, and histological alterations. Moreover, the study highlighted the beneficial effect of blocking the 5-HT3 receptor by using palonosetron and, to a lesser extent, the α7nAChR by using MLA to attenuate AD-associated alterations via interfering with the interconnection between activated microglia/inflammasome/Aβ and the depleted ACh and 5-HT. The NLRP3 inflammasome is definitely an important therapeutic target for delaying AD progression, and it could be speculated that using both 5-HT3 receptor and α7 nAchR antagonists can combat AD pathology and avert AD progression more effectively than either alone.

## Data Availability

Not applicable.
